# Characterization of phospholipid vesicles containing lauric acid: physicochemical basis for process and product development

**DOI:** 10.1016/j.heliyon.2019.e02648

**Published:** 2019-11-01

**Authors:** Laura Farkuh, Paulo T. Hennies, Cláudia Nunes, Salette Reis, Luisa Barreiros, Marcela A. Segundo, Pedro L. Oseliero Filho, Cristiano L.P. Oliveira, Alexandre Cassago, Rodrigo V. Portugal, Rodrigo A. Muramoto, Gustavo P.B. Carretero, Shirley Schreier, Hernan Chaimovich, Iolanda M. Cuccovia

**Affiliations:** a Department of Biochemistry, Institute of Chemistry, University of São Paulo, São Paulo, Brazil; b Líbera Tecnologia e Inovação Ltda., São Paulo, Brazil; c LAQV, REQUIMTE, Faculty of Pharmacy, University of Porto, Porto, Portugal; d Department of Experimental Physics, Institute of Physics, University of São Paulo, São Paulo, Brazil; e Brazilian Nanotechnology National Laboratory (LNNano), Brazilian Center for Research in Energy and Materials (CNPEM), Campinas, Brazil

**Keywords:** Biotechnology, Pharmaceutical Science, Cryo-TEM, Lauric acid, DSC, Lipid Vesicles, DLS, Ionizable solute, SAXS

## Abstract

Lauric acid (LAH) strongly inhibits the growth of acne-causing bacteria. LAH is essentially water-insoluble and the solubility of laurate (LA) salts are medium and temperature dependent. Hence, LAH/LA preparations are difficult to formulate. Here we fully characterized phospholipid vesicles containing up to 50 mol% LAH. Vesicles of dipalmitoylphosphatidylcholine (DPPC) containing LAH, at pHs 7.4 and 5.0, were characterized measuring size, charge, bilayer phase transition temperature (Tm) and permeability of water-soluble probes. Small angle X-ray scattering and *cryotransmission electron microscopy* showed multilamellar vesicles at low LAH %. Increasing LAH % had a negligible effect on particle size. An internal aqueous compartment in all vesicle's preparations, even at equimolar DPPC: LAH fractions, was demonstrated using water-soluble probes. At pH 5.0, the interaction between DPPC and LAH increased the Tm and phase transition cooperativity showing a single lipid phase formed by hydrogen-bonded DPPC: LAH complexes. At pH 7.4, vesicles containing 50 mol% LAH exhibited distinct phases, ascribed to complex formation between LAH and LA or LAH and DPPC. LAH incorporated in the vesicles minimally permeated a skin preparation at both pHs, indicating that the primary sites of LAH solubilization were the skin layers. These results provide the foundations for developing processes and products containing DPPC: LAH.

## 1 Introduction

The inhibition of bacterial growth by free fatty acids (FFA's) is well established [1]. Lauric acid (LAH), the saturated fatty acid with the highest antimicrobial potential, found in coconut (oil and milk), is also present in small amounts in sebum [2, 3, 4]. LAH inhibits the growth of *Propionibacterium acnes*, *Staphylococcus aureus*, and *Staphylococcus epidermis*, and, therefore, LAH could treat acne vulgaris [5]. LAH is poorly soluble in water, and the solubility of laurate (LA) depends critically on the counterion and the solution temperature [6, 7, 8]. The water solubility properties of LAH and LA are significant drawbacks for the use of this antimicrobial agent in pharmaceutical formulations.

Phospholipid vesicles are efficient drug carriers [9] and both LAH and LA intercalate in their bilayers, changing structure and interfering in the membrane permeability to water-soluble compounds [10,11]. Understanding the fundamental properties of these aggregates is essential to allow the applicability of these formulations to treat infectious diseases.

The mixture of phospholipids vesicles and fatty acids can lead to changes in the vesicle's membrane intrinsic structure because the presence of fatty acids can disrupt the well-organized phospholipid bilayer. As fatty acids dissociate, the pH of the environment can also modify the membrane properties due to different ratios of charged/neutral forms of the carboxylic group. Addition of fatty acids to pre-formed dimyristoyl phosphatidylcholine (DMPC) vesicles changes both turbidity and the phase transition temperature (Tm) [9]. Release of DMPC vesicle-entrapped fluorescent probes accompanies the turbidity changes, demonstrating the disrupting effect of externally added fatty acids on the bilayer structure [11]. Despite the disruptive effect of fatty acids on phospholipid membranes, some fatty acids, such as oleic acid in the pH range 8–9, form multilamellar vesicles with bilayers stabilized by hydrogen bonding between the protonated and anionic forms [12]. These seemingly diverse actions of fatty acids make the study of these phenomena necessary.

The present study focuses on the physicochemical properties of large unilamellar vesicles (LUVs) prepared with dipalmitoylphosphatidylcholine (DPPC): LAH mixtures. The effect of LAH on the properties of vesicles prepared with variable proportions of dipalmitoylphosphatidylcholine, DPPC, was studied by mixing the fatty acid with DPPC before vesicles preparation, at different DPPC: LAH molar ratios and different pHs. This method allowed the formation of vesicles where the phospholipid bilayers are already in its equilibrium state. We analysed the bilayer structure of these mixed vesicles, as well as the Tm, size, charge, and permeability of water-soluble probes. Additionally, the delivery of lauric acid from the vesicles to a skin preparation was studied. Our results may provide the foundations for developing processes and products containing DPPC: LAH.

## 2 Materials and methods

### 2.1 Materials

1,2-Dipalmitoyl-*sn*-glycero-3-phosphocholine (DPPC) [Avanti Polar Lipids (Alabaster, USA)]; lauric acid (LAH), l-ascorbic acid, tris (hydroxymethyl) aminomethane (Tris), polyoxyethylene-9-lauryl ether (polydocanol), Sephadex G-25 medium, acetic acid, chloroform, and methanol [Sigma Aldrich (St. Louis, USA)] were used as received. 5(6)-Carboxyfluorescein (CF) [Sigma Aldrich] was purified and stocked as the sodium salt [13]. The probes 4-trimethylammonium-2,2,6,6-tetramethylpiperidine-1-oxyl iodide (CAT1) and tetrasodium 1,3,6,8-pyrenetetrasulfonate (PTS) [Molecular Probes (Eugene, USA)] were used as received.

For LC-MS/MS analysis, acetonitrile (LiChrosolv LC-MS grade) was from Merck (Darmstadt, Germany) and water was from purification system (resistivity > 18 MΩ cm, Sartorius, Göttingen, Germany). Mobile phase components were filtered through 0.45 μm Millipore (Billerica, MA) HVHP and 0.22 μm Millipore GVWP filters, respectively, and degassed in an ultrasonic bath for 15 min.

### 2.2 Lauric acid quantification (LC-MS/MS analysis)

LAH concentration was determined by liquid chromatography coupled to triple quadrupole tandem mass spectrometry (LCMS-8040) equipped with an electrospray ionization source (ESI) (Shimadzu Corporation).

### 2.3 Liposome preparation

Stock solutions of DPPC 0.05 M and LAH 0.05 M in chloroform: methanol 3:2 (v/v) were prepared. The stock solution of DPPC was quantified by determining the phosphate concentration of the sample [13] and the concentration of the LAH solution was taken by weight. Lipid mixtures, at the desired molar ratio of DPPC: LAH, were prepared by mixing aliquots of the stock solutions into glass tubes. The following lipid compositions were used: 100 % DPPC; DPPC: LAH 90:10; DPPC: LAH 80:20; DPPC: LAH 70:30; DPPC: LAH 60:40 and DPPC: LAH 50:50. The solvents were eliminated under an N_2_ flux forming a film and solvent traces were removed in a vacuum chamber (2 h). Multilamellar vesicles (MLVs) were prepared by adding the desired solution to the lipid film and vortexing the mixture until the film detached from the tube wall. Large unilamellar vesicles (LUVs) were obtained by extruding MLVs suspensions in an Avanti mini-extruder, Alabaster, USA, using two membranes with 100 nm pore size (eleven passages) at 60 °C.

### 2.4 Dynamic light scattering (DLS)

The LUVs hydrodynamic diameter (D_H_), the polydispersity index (PdI), zeta potential and the number of particles were determined by Dynamic Light Scattering (DLS), in triplicate, at room temperature using a Zetasizer Nano ZS, Malvern Instruments Ltd.

### 2.5 Small angle X-ray scattering (SAXS)

Small angle X-ray scattering (SAXS) spectra were obtained with a Bruker Nanostar (with optimized optics by Xenocs) at 25 °C. DPPC: LAH vesicles (10 mM total lipid) in 50 mM Tris-HCl buffer, pH 7.4, or 50 mM Na acetate, pH 5.0 were placed in 1.5 mm diameter glass capillaries. The experimentally accessible range of the modulus of the transfer moment vector, q=4πsin(θ)/λ (where θ is half the scattering angle and λ the X-ray wavelength), was $0.018\;{Å}^{-1}-\;0.35\;{Å}^{-1}$. SUPERSAXS program [14] was used for data treatment, allowing correction of the raw data using background scattering, empty capillary and sample transmission, and then normalization to absolute scale using water as standard. The final data correspond to one-dimensional scattered intensity I(q), with respective uncertainties, versus q values.

### 2.6 Cryotransmission electron microscopy (Cryo-TEM)

For the cryogenic transmission electron microscopy (Cryo-TEM) analysis, DPPC: LAH 70:30 and DPPC: LAH 50:50 (mole: mole) vesicles were used at 2.5 mM total lipid in 10 mM Tris-HCl, pH 7.4, or 10 mM Na acetate, pH 5.0, buffers. A 3 μL droplet was deposited on a 300 mesh lacey carbon-coated copper grid (TED Pella) using an easyGlow discharge system (Pelco) with 15 mA negative current for 10 s. Specimens were prepared at 22 °C and humidity 100 % in an automated vitrification system (Vitrobot Mark IV, FEI, The Netherlands) with a blot time of 3 s, blot force of 0, and 20 s waiting time before blotting. Specimens were analyzed in low dose condition, with a defocus range of −2 μm to −4 μm, using a Jeol JEM-2100 (Pleasanton, USA) microscope equipped with an F-416 CMOS camera (TVIPS, Germany), operating at 200 kV.

### 2.7 Differential scanning calorimetry (DSC)

Phase transition temperature (Tm) and cooperativity (ΔT^1/2^) were determined on a MicroCal VP-DSC microcalorimeter, Malvern Instruments Ltd. (Malvern, UK). Demineralized water was used as a reference. The data were analyzed with the Origin 8.5 program.

### 2.8 Entrapment of hydrophilic probes

#### 2.8.1 Fluorescent probes

Solutions (500 μL) containing 2 mM CF in 50 mM Tris-HCl buffer, pH 7.4, or 1 mM PTS in 50 mM Na acetate buffer, pH 5.0, were added to DPPC: LAH films (10 mM total lipid concentration), and the resulting MLVs were extruded.

A Sephadex G25 column (1.5 cm diameter, 45 cm height) was used to separate CF- or PTS-containing vesicles from the non-encapsulated fluorescent probes. The sample (300 μL) was applied to the column, eluted with the desired buffer, and 1 mL fractions were collected. The percentage of CF (or PTS) encapsulation was calculated according to Eq. (1):

(1) $$\%\;encapsulation=\;100*\;\frac{moles\;Vo}{moles\;Vo+moles\;Vi}\;$$

where Vo and Vi are the excluded and internal column volumes respectively and *moles Vo* and *moles Vi* are the number of moles eluted at *Vo* and *Vi*, respectively. Fluorescence was measured using a Shimadzu RF-5301PC fluorimeter, with λ_exc_ 490 nm, λ_em_ 520 nm for CF, and λ_exc_ 355 nm, λ_em_ 405 nm for PTS [15]. For the fractions containing vesicles, fluorescence were measured after the addition of 25 μL of an aqueous solution of 10% polidocanol (v/v) to eliminate vesicle-produced light scattering.

#### 2.8.2 Electron paramagnetic resonance (EPR) probe

CAT1 solutions (3.75 mM) were prepared in 50 mM Tris-HCl, pH 7.4, or 50 mM Na acetate, pH 5.0. Ascorbic acid (0.04 M), prepared in the same buffers, was used to suppress the CAT1 signal [16, 17, 18].

DPPC: LAH films were hydrated with CAT1 solutions (10 mM lipid), vortexed and extruded. Vesicles with CAT1 (170 μL) were placed in flat quartz cells (Wilmad, USA) and the EPR spectrum was recorded (*total signal*); then, 10 μL of sodium ascorbate were added, supressing CAT1 signal outside the liposomes (*final signal*). Controls assured that ascorbate concentration was sufficient to reduce external CAT1.

EPR spectra were acquired at room temperature on a Bruker EMX-200 spectrometer with 5 mW microwave power, 1 G modulation amplitude, sweeping a scan range of 16 G with the field centered at 3455 G. The gain was adjusted according to the sample concentration. Spectra were analyzed with the WINEPR software (Bruker), and the peaks were integrated twice to obtain the area, which is proportional to the probe concentration. The percentage of encapsulated CAT1 was calculated according to Eq. (2):

(2) $$\%\;encapsulated=100\;\times \;\frac{final\;signal}{total\;signal}$$

### 2.9 Lauric acid skin permeation

The skin permeation of LAH was studied using a vertical static Franz diffusion cells with pig ear skin as membrane. Skin was cleaned, the hypodermis removed and stored at -20 °C until its use. Five ml buffer were placed in the receptor chamber, and 500 μL of sample (0.5 mM total lipid) or buffer solution (control) in the donor chamber. A 500 μL aliquot of solution from the receptor chamber (37 °C) was taken every 1 hour (for 8 h) to quantify LAH. To maintain sink conditions, 500 μL of buffer solution was replenished in the receptor chamber. The skin permeation (μg/cm^2^) was calculated dividing the amount of LAH in the acceptor chamber per skin area (0.64 cm^2^). The sample was transferred to a tube, 0.2 mL of HCCl_3_ was added and LAH was extracted. This process was repeated 3 times. The solvent was evaporated with an N_2_ flux at 45 °C and the LAH was solubilized in 500 μL of a mixture of acetonitrile:water 80:20 v/v. From each sample, three injections were made into the HPLC and the data in Fig. 6 are averages.

Chromatographic analysis were performed in a Nexera X2 UHPLC system comprising two LC-30AD pumps, a DGU-20A5R degassing unit, a SIL-30AC autosampler, and a CTO-20AC oven (Shimadzu Corporation, Kyoto, Japan).

Chromatographic separation was achieved using a reversed phase Kinetex core-shell C8 column (150 mm × 2.1 mm, 2.6 μm particle size; Phenomenex, Torrance, CA, USA), at 30 °C. Elution was performed in an isocratic mode using acetonitrile:water (80:20, v/v) at a flow rate of 0.2 mL min^−1^. The injection volume was 2 μL and total analysis time was 12 min.

The mass spectrometer was operated in negative ionization mode (ESI-), and data were acquired using multiple reaction monitoring (MRM) mode to enhance selectivity. The *m/z* transition 199.30 > 181.25, corresponding to the neutral loss of one water molecule from the molecular ion [M-H]^-^, was used for quantification and identification purposes. The nebulizing gas (N_2_) flow rate was 3 L min^−1^, desolvation line temperature 250 °C, heat block temperature 400 °C, detector voltage, 1.76 kV and collision gas (argon) 230 kPa. For calibration, LA standard solutions were between 0.1 and 10 μM in the mobile phase. Peak detection and quantification were performed using LabSolutions software version 5.60 SP2 (Shimadzu Corporation).

## 3 Results and discussion

### 3.1 Small angle X-ray scattering (SAXS)

**Fig. 1:**
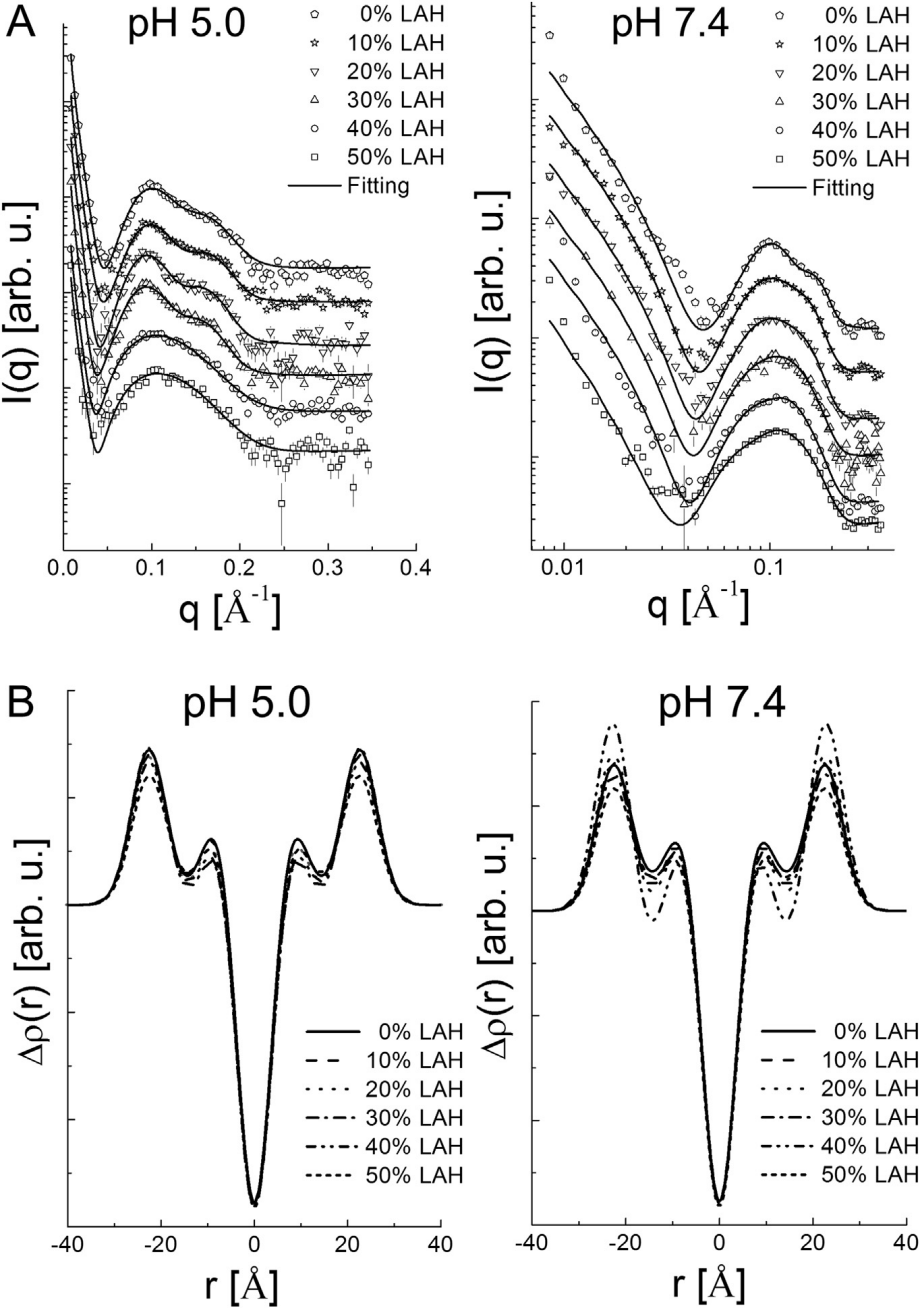
(A) Experimental data (open symbols) of electron density contrast profile for DPPC: LAH vesicles with increasing LAH content at pH 5.0 and 7.4. Solid lines are fittings using the Gaussian Deconvolution Method. (B) Electron density contrast profile obtained from fittings of the experimental SAXS data at pH 5.0 and 7.4, at 10 mM total lipid concentration.

Fig. 1 shows SAXS data for vesicles with different DPPC: LAH molar ratios and at different pHs. The treated data of electron density contrast profile in Fig. 1A (open symbols), were characteristic of uni- or multilamellar vesicles. The main difference between them was related to the MLV exhibiting an oscillation on the bump in the region $0.06\;{Å}^{-1}<q<0.2\;{Å}^{-1}$, indicating that the number of correlated lipid bilayers (N) was >1. For instance, considering Fig. 1A, pH 5.0, the curves corresponding to 40 % and 50 % LAH are typical of unilamellar vesicles, since there was no oscillation on the bump in the $0.06\;{Å}^{-1}<q<0.2\;{Å}^{-1}$ regions, differently from the remaining curves, typical of MLV.

DPPC vesicles, at pH 5.0 and pH 7.4, were multilamellar (Fig. 1A). Since the overall shapes of the SAXS curve were similar, the vesicles exhibited similar structures in this qrange. Increasing the amount of LAH to 10 %, a decrease of the multilamellar vesicle population was observed at pH 7.4. The same transition was observed at pH 5.0 only above 40 % LAH. This result indicates, qualitatively, the importance of pH on the nanostructure of this system. The fact that vesicles have a greater tendency to be unilamellar at pH 7.4 is probably due to the negative charge imparted on the surface by the negatively charged laurate ion (LA), which promotes inter-bilayer repulsion, leading to a decrease in the number of lamellae.

**Scheme 1:**
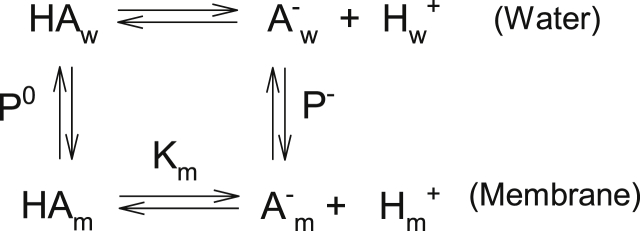
Water/membrane partitioning of a weak acid (HA) and its anion (A^−^). The indexes w and m refer to water and membrane respectively, P^0^ and P^−^ are the partitioning coefficients of HA and A^−^. K_m_ and K_w_ are the dissociation constants of HA in the membrane and in water.

The clear difference between the effects of LAH and LA on the resulting structures bring to attention that few investigations on binding of ionizable solutes to biological and model membranes consider that partitioning of the charged and uncharged forms of these compounds differs, the uncharged form usually incorporating to a more significant extent. In Scheme 1, HA and A^**-**^ are the uncharged and anionic forms of the solute, respectively, K_w_ and K_m_ are the ionization constants in the aqueous and membrane phases, respectively, and P^0^ and P^**-**^ are the partition coefficients of both forms.

P is defined as:

(3) $$P=\frac{n_{m}.V_{w}}{n_{w}.V_{m}}$$

where n and V represent the number of moles and volume, respectively, and the indexes m and w the membrane and aqueous phases, respectively. Thus, pK_w_ and pK_m_ are, respectively, the pHs of the bulk aqueous phase where the populations of charged and uncharged forms *in the aqueous phase* are equal and where the populations of charged and uncharged forms *in the membrane* are equal. ΔpK (pK_m_ – pK_w_) is a necessary consequence of the fact that P^0^ ≠ P^**-**^.

This analysis was applied in studies of ionizable compounds binding to micelles [19] and lipid vesicles [20]. An apparent pK, pK_app_, was defined, corresponding to the aqueous phase pH where the population of the charged species equals the population of the uncharged species, both being the sum of their fractions in the membrane and the aqueous phase and depend on membrane concentration.

The apparent pK of carboxylic acids in membranes significantly increases, and the pK_w_ of the free acid, around 5.0, increases to *ca*. 7.0–7.5 [21,22]. The membrane-bound fraction will depend on total membrane concentration. For LA, at pH 7.4, the total partitioning of LA into the membrane will decrease due to the contribution of both P^0^ and P^−^ to P_av_, and the much lower value of P^−^. At pH 5.0 essentially all LAH is protonated and fully incorporated in the membrane, as the partition coefficient (P^0^) is sufficiently high.

To obtain quantitative information of overall structural changes in the system, the experimental SAXS curves were fitted using the Gaussian Deconvolution Method [14], which allows simultaneous determination of both form and structure factor. Since the form factor is calculated from the modeled electron density contrast profile, Δρ(r), it is possible to detect structural bilayer changes either in Δρ(r)or in bilayer thickness (δ) due to the presence of LAH. Also, from the structure factor, it is possible to obtain information about membrane flexibility (through the Caillè parameter, (η)), lamellar periodicity (D) and the number of correlated bilayers (N). The fittings shown in Fig. 1A (solid lines) describe the behavior of the experimental data satisfactorily. These results include the modeled electron density contrast profile of the lipid bilayers, shown in Fig. 1B, and the values of the other parameters, summarized in Table 1. It is important to note that the values shown in the table are average values. Thus, N = 1.3 ± 0.1 represents the average number of bilayers in a large vesicle ensemble. For vesicles with N close to 1 (considering error bars), *i.e*., the vesicles were unilamellar, structure factor parameters cannot be evaluated, and "NA" in Table 1 refers to not applicable.

**Table 1:** SAXS parameters obtained from fittings of experimental curves for DPPC vesicles with variable LAH content at pH 5.0 and 7.4 (Fig. 1A) using the Gaussian Deconvolution Method. For unilamellar vesicles (N close to 1), structure factor parameters (*D* and *η*) are not applicable (“NA”).

pH	% LAH	Parameter			
		D(Å)	δ(Å)	N	η
5.0	0	68.0 ± 9.1	51.2 ± 4.9	1.3 ± 0.1	0.04 ± 0.05
	10	68.9 ± 1.1	50.8 ± 5.1	1.3 ± 0.1	0.02 ± 0.01
	20	70.3 ± 0.2	52.5 ± 9.2	1.4 ± 0.1	0.26 ± 0.10
	30	69.9 ± 9.1	51.4 ± 6.9	1.3 ± 0.2	0.23 ± 0.03
	40	NA	52.0 ± 1.9	1.0 ± 0.1	NA
	50	NA	50.7 ± 5.9	1.0 ± 0.1	NA
7.4	0	67.7 ± 7.8	52.1 ± 3.2	1.3 ± 0.2	0.08 ± 0.02
	10	NA	52.0 ± 6.9	1.1 ± 0.1	NA
	20	NA	52.6 ± 2.7	1.0 ± 0.1	NA
	30	NA	50.8 ± 3.7	1.0 ± 0.1	NA
	40	NA	50.9 ± 0.7	1.1 ± 0.1	NA
	50	NA	51.3 ± 5.1	1.1 ± 0.1	NA

The electron density contrast profile (Fig. 1B) is related to the lipid bilayer hydrophobic (central part of the curve) and hydrophilic (outer parts of the curve) regions. The profiles had the same overall aspect in all cases, but significant changes were found in the hydrophilic part, due to the presence of LAH, demonstrating an interaction between the fatty acid and the hydrophilic part of the lipid bilayer. The electron density changes were more pronounced at pH 7.4, (Fig. 1B) indicating that negatively charged LA exerts a significant effect on the bilayer structure.

Within experimental error, DPPC-LAH interactions did not change membrane thickness to a considerable extent, as demonstrated by the values of δ close to 50Å at both pHs and all DPPC: LAH ratios (Table 1). The results for N confirmed multi- to unilamellar transitions, as mentioned above. At pH 5.0, the multi- to unilamellar transition was evident at 40% LAH. At pH 7.4, the transition was apparent from 10% LAH (Table 1). At pH 7.4, it is important to note that since the bump in the $0.06\;{Å}^{-1}<q<0.2\;{Å}^{-1}$ region exhibits a slight oscillation for curves related to 10 %, 20 %, 30 %, 40 %, and 50 % LAH, the value of N obtained from the fitting is not precisely 1.0 because in all cases, uni- and oligolamellar vesicles coexist, especially when N is low.

As observed for membrane thickness, the lamellar periodicity, estimated by the *D* parameter (for multilamellar vesicles) remained approximately constant (70Å, within experimental error). On the other hand, the Caillè parameter η ​ increased as the amount of LAH increased, at both pHs, indicating that the lipid membrane became more flexible.

### 3.2 Cryo-transmission electron microscopy (Cryo-TEM)

**Fig. 2:**
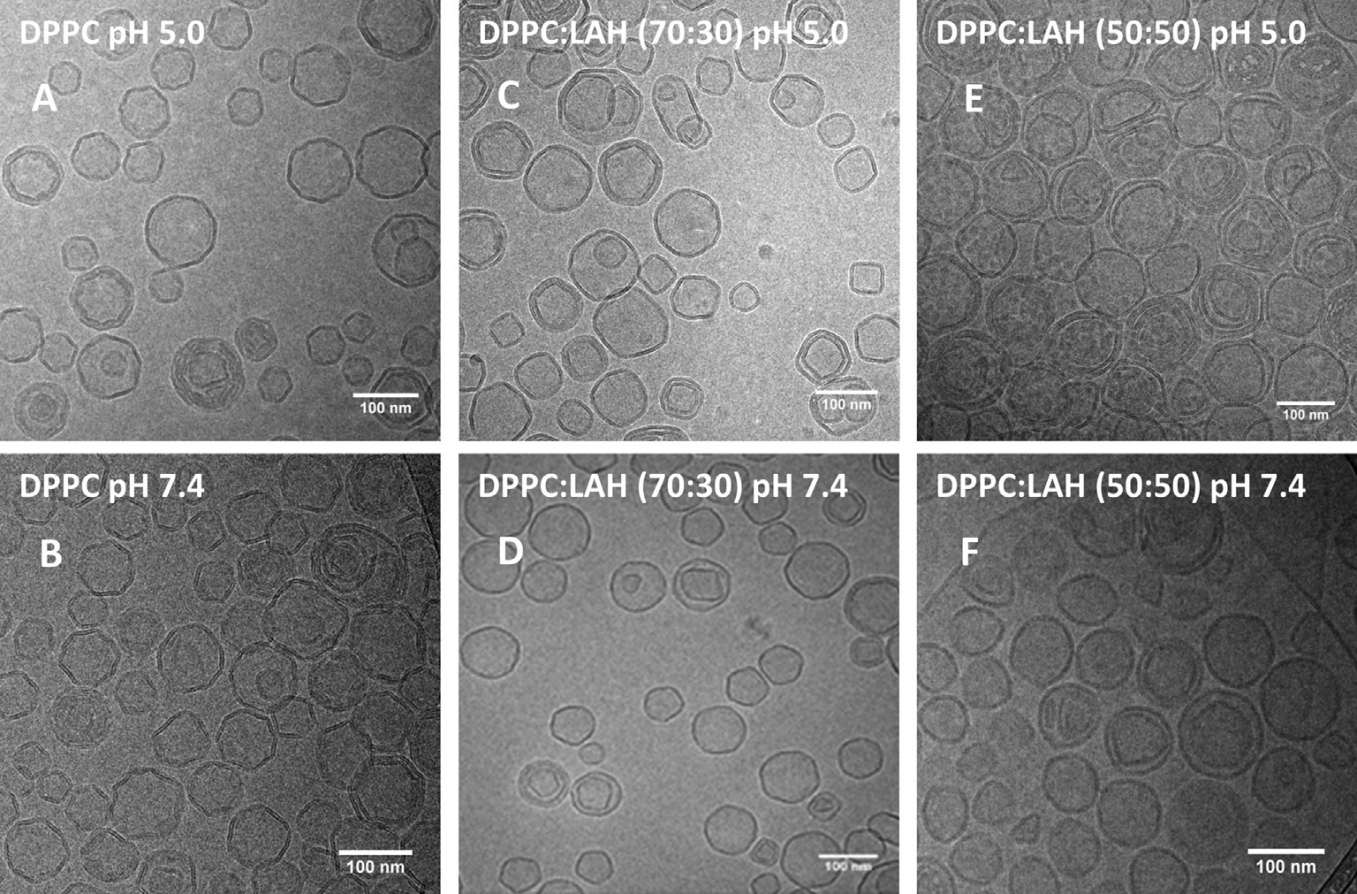
Cryo-TEM images of vesicles of: (A,B) pure DPPC; (C,D) DPPC: LAH 70:30 and (E,F) DPPC: LAH 50:50 in 10 mM Na acetate pH 5.0, and 10 mM Tris-HCl pH 7.4. Total lipid concentration was 2.5 mM.

Micrographs of pure DPPC vesicles exhibited polygonal shapes, both at pH 5.0 and pH 7.4 ​(Fig. 2, A, B), similarly to dimyristoylphosphatidylcholine [23, 24]. Even though the vesicles were extruded using 100 nm pore size membranes, the micrographs indicated that oligolamellar and small vesicles (<100 nm in diameter) were also present (Fig. 2), as described using cryo-TEM data for DMPC [25]. For vesicles containing 30% LAH at pH 5.0 (Fig. 2C) and 7.4 (Fig. 2D), vesicles with two bilayers and a smaller quantity of multilamellar vesicles were observed (Fig. 2, C, D), in reasonable agreement with the SAXS data (Table 1), that showed that the increase in LA favored the formation of unilamellar vesicles. Besides, at this condition, some square-like vesicles and other non-spherical structures were observed (Fig. 2, C, D). Some of these shapes could be due to the existence of LA-enriched regions.

Samples containing 50% LAH showed more spherical vesicles and some membrane fragments (Fig. 2, E, F). A further increase the LAH content causes the bilayer to become more homogeneous, displaying a smaller proportion of domains. The images clearly show that different DPPC/LAH mixtures formed vesicles at both pHs (Fig. 2).

### 3.3 Determining average particle size by Dynamic Light Scattering (DLS)

**Fig. 3:**
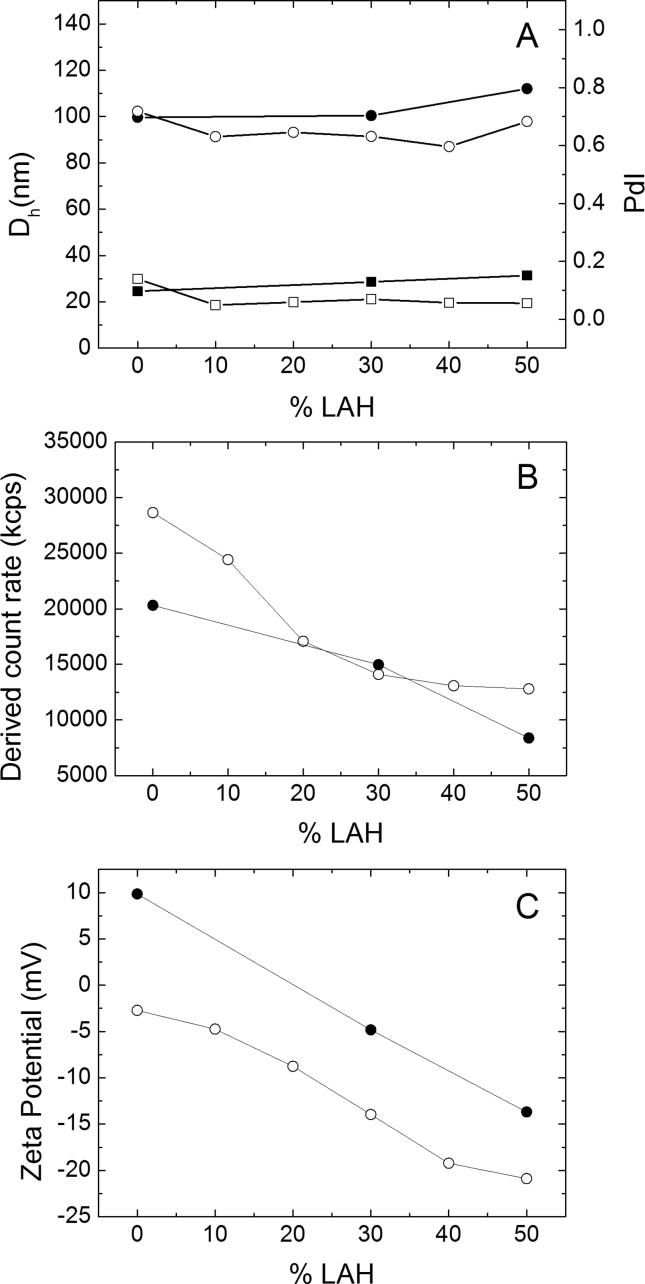
Dynamic Light Scattering and Zeta Potential of LUV's of DPPC: LAH (0.5 mM) with different %LAH in 10 mM Tris-HCl, pH 7.4 (○, □) and 10 mM Na acetate, pH 5.0 (●, ■). (A) D_H_ (○, ●) and PdI (□, ■); (B) Derived Count Rate (kcps) (○, ●) and (C) Zeta potential (○, ●).

The addition of increasing amounts of LAH to DPPC vesicles had little effect on the apparent hydrodynamic diameter, D_H_ (Fig. 3A). The vesicle size was mainly determined by the pore size of the extrusion membrane [26]. At 0.5 mM total lipid vesicle's diameters varied in the range 100 ± 10 nm for all lipid compositions both at pH 5.0 and 7.4 (Fig. 3A). The polydispersity index (PdI) was low (<0.2) for all preparations (Fig. 3A) indicating the homogeneity of these samples. The cryo-TEM micrographs also did not suggest considerable changes in particle size with LAH: DPPC ratios, though the latter studies were performed with 2.5 mM total lipid (Fig. 2).

Since the extrusion membrane pore size determined vesicle size, the replacement of DPPC by LAH should lead to a change in the total number of lipid molecules (DPPC + LAH) in each vesicle. The surface area of the outer vesicle monolayer (50 nm radius) is 31.4 × 10^3^ nm^2^; assuming a bilayer thickness of 5.0 nm, the surface area of the inner monolayer is 25.4 × 10^3^ nm^2^, yielding a total of 56.8 × 10^3^ nm^2^. Considering that the cross-sectional area of DPPC in the gel phase is *ca.* 0.49 nm^2^ [27], one can calculate that a vesicle with D_H_ = 100 nm will accommodate 116 × 10^3^ DPPC molecules. On the other hand, the cross-sectional area of LAH can be taken as 0.22 nm^2^ [28,29]. Thus, in samples where LAH replaces DPPC molecules, a higher total number of (DPPC + LAH) molecules will occupy the same vesicle total surface area. As a result, if the total lipid concentration is the same, a smaller number of vesicles of the same size will be formed. It can be estimated that 16 % less with respect to pure DPPC vesicles should be formed in the case of samples containing 30 % LAH and that 30 % fewer vesicles should be formed in the case of 50 % LAH-containing samples. This analysis applies to pH 5.0 when LAH is fully incorporated.

As the diameter of the vesicles prepared at all DPPC: LAH ratios were similar and had the same total lipid concentrations, the derived count rate (DCR) of the samples, at both pHs, can be compared, and were proportional to the number of vesicles. The decrease in DCR with LAH was more significant at pH 7.4 than at pH 5.0 (Fig. 3B). Oligolamellar vesicles can account for a decrease in DCR higher than that predicted. This decrease can also be due to the higher partition of LA in the aqueous phase at pH 7.4 than pH 5.0, leading to a decrease in the number of vesicles. As expected, under all conditions, vesicles of DPPC: LAH prepared at pH 5.0 exhibited a less negative zeta potential than those prepared at pH 7.4 (Fig. 3C).

### 3.4 Estimation of the internal vesicle's volume by fluorescent and spin label probes

LUVs were prepared at different DPPC: LAH molar ratios, and three different water-soluble probes were entrapped in the LUVs to check the bilayer permeability as a function of pH.

**Fig. 4:**
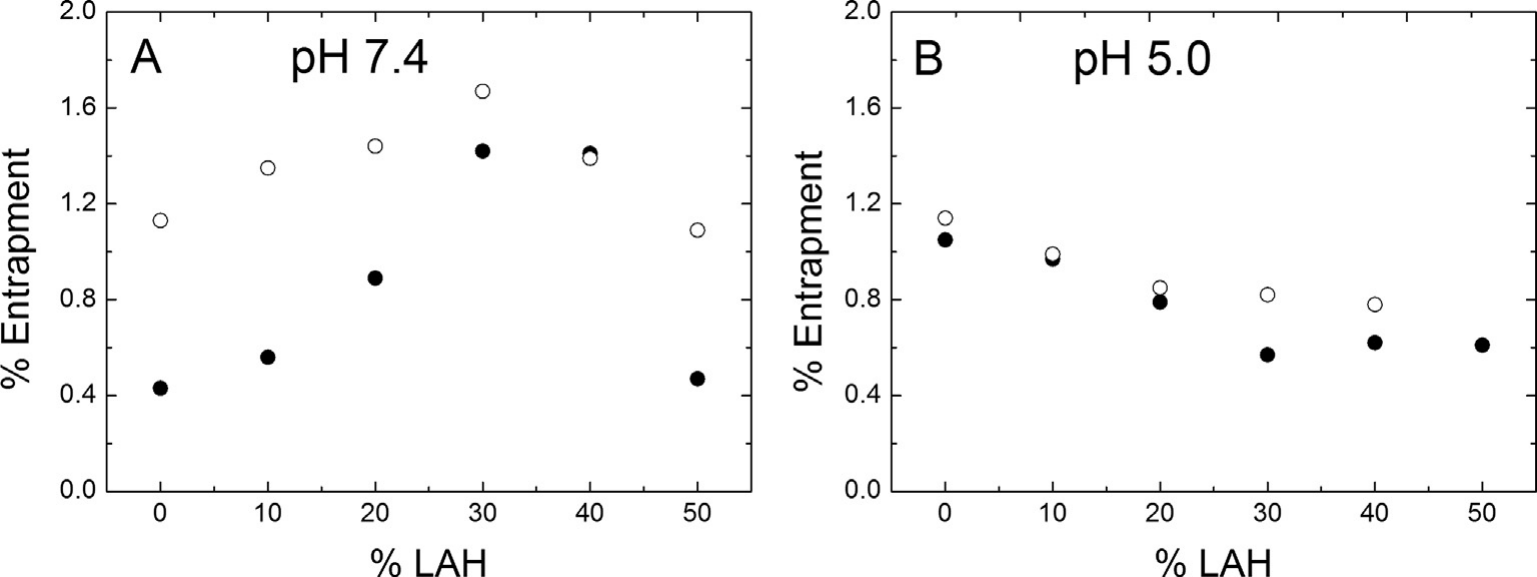
Entrapment of probes in DPPC vesicles as a function of LAH content. Total lipid concentration 10 mM. (A) (●) CF and (○) CAT1 in 50 mM Tris-HCl buffer, pH 7.4. (B) (●) PTS and (○) CAT1 in 50 mM Na acetate buffer, pH 5.0.

Vesicles retained CF, CAT1 and PTS, demonstrating that DPPC: LAH vesicles containing up to 50% LAH possessed an internal aqueous compartment, both at pH 7.4 and 5.0 (Fig. 4). At pH 7.4 CF entrapment increases from 0.4 % in pure DPPC, to almost 1.2 % in 30 % LA, decreasing with further LAH increase. When the probe used was CAT1, the % entrapment, at pH 7.4, was almost 1.2 % for all DPPC: LAH molar fractions. These results show that all vesicles have a relative impermeability to water soluble compounds and that the probe structure characteristics interfere in the % entrapment of the vesicles. As CF is insoluble at low pH, PTS was used at pH 5.0 (see Methods). For pure DPPC, considering the molecule's and the vesicle's geometry described above, we calculate that the ensemble of vesicles should retain 1.9 % of the total aqueous volume, at 10 mM total lipid concentration. At pH 5.0 both probes, PTS and CAT1 entrapped 1.2 % of the total volume (Fig. 4B), and the same value was obtained for CAT1 at pH 7.4 (Fig. 4A). We ascribe the differences between the theoretical and experimental results to some permeability of the probes due to their capability to protonate and then permeate the membrane and to the presence of a population of oligolamellar vesicles.

As discussed above, increasing LAH should lead to a decrease in the number of vesicles, and, therefore, a decrease in the trapped volume. The calculated volume should decrease to 1.0 % and 0.84 % for vesicles containing 30 % and 50 % LA, respectively. Comparing the entrapment of CAT1 and PTS at pH 5.0, it is observed that both probes yielded similar results at almost all LAH contents, the entrapment decreasing to *ca.* 0.6 % (for PTS) and 0.85 % (for (CAT1) at 30 mole % LAH and to 0.7 % (for PTS) at 50 mole % LAH (Fig. 4B).

As LUVs containing different DPPC: LAH ratios retained water soluble compounds, besides the difference in structures of DPPC and LAH, all formulations preserved the aqueous compartment from leakage. The efficiency of encapsulation of phosphatidylcholine liposomes varies with the addition of charged amphiphilic compounds to the membrane composition [18]. At pH 7.4 (Fig. 4A), condition where approximately [LAH = LA], the same behavior is evident, with an increase in the entrapment percentage as the LAH amount in the membrane is increased up to 30 mol%; from this point on, at 40 and 50 mol% of LAH, the formation of a smaller number of 100 nm LUV, as discussed in the DLS results section, decreases the total entrapment of CAT1 or CF. At pH 5.0 (Fig. 4B), with all LAH molecules in the membrane found in the neutral form, no charge effect takes place in the entrapment efficiency and only a decrease in the total entrapment, related to the formation of a smaller number of vesicles, is observed.

### 3.5 Looking at lipid organization with differential scanning calorimetry (DSC)

**Fig. 5:**
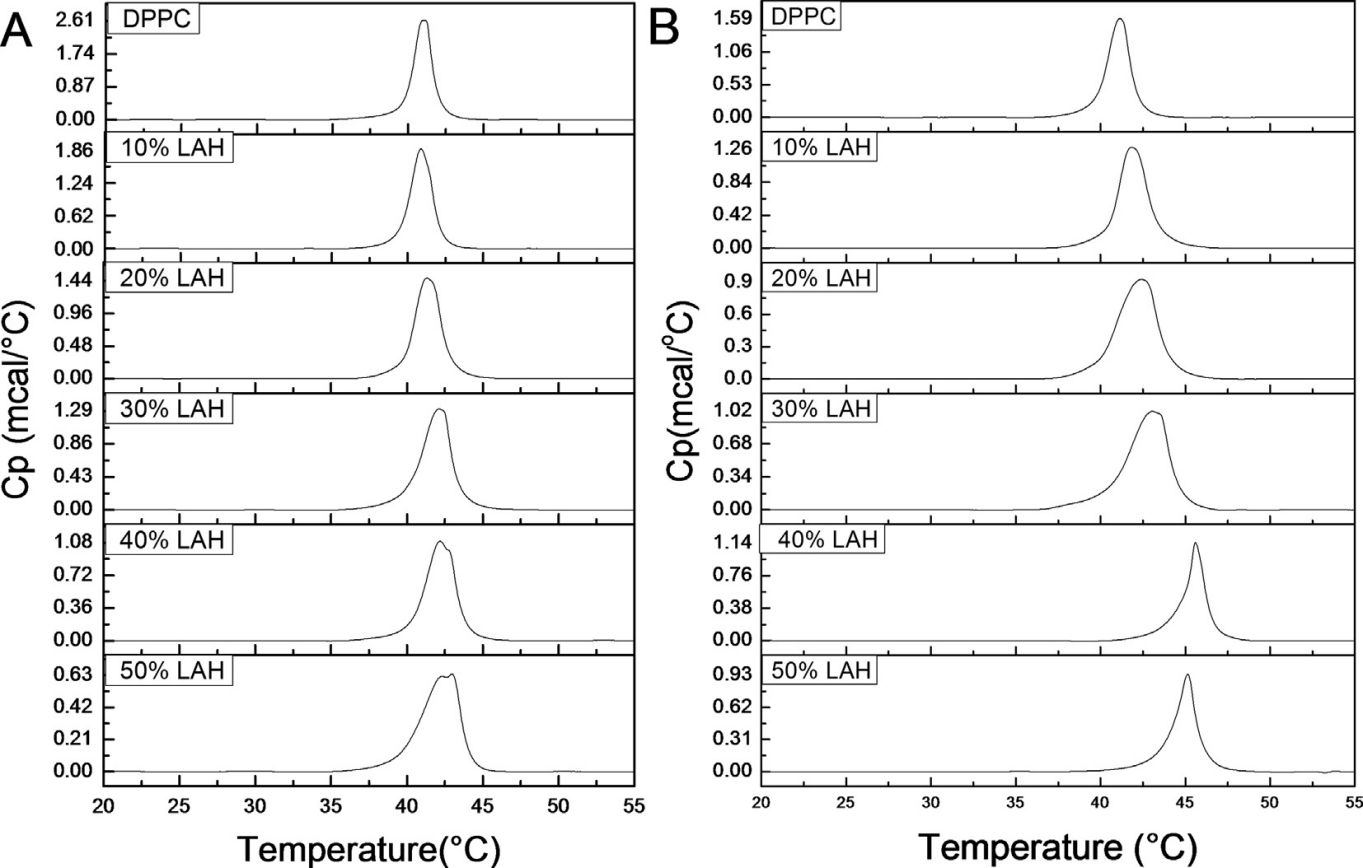
Thermograms of DPPC: LAH vesicles with increasing LAH content. (A)10 mM Tris-HCl, pH 7.4 buffer and (B) 10 mM Na acetate buffer, pH 5.0. Total lipid concentration, 1 mM.

At pH 7.4, the T_m_ of vesicles increased with LAH (Fig. 5A, Table 2). At 10 % LAH, a shoulder appears at a higher temperature, and the width of the peaks increased with LAH (Fig. 5A). At 40 % and 50 % LAH, two superimposed components are seen in the thermogram. Tm and ΔT^1/2^ for all DPPC: LAH mixtures at pHs 5.0 and 7.4 are in Table 2. For 40 % and 50 % DPPC: LAH vesicles at pH 7.4, two values of Tm can be calculated from Fig. 3, and both values are given in Table 2. Such behavior could be due to the heterogeneity of vesicle populations even for pure phospholipid vesicles [25]. In the present case, at pH 7.4, both LAH and LA may incorporate in the bilayer, and each species may interact with phospholipids differently. Protonated long chain fatty acids form strong complexes with phosphatidylcholine phospholipids, leading to increases in the phase transition temperature [30, 31, 32, 33], most likely due to hydrogen bond formation between the fatty acid carboxyl –OH group and the phospholipid phosphate group. It is reasonable to expect strong hydrogen bonding at the membrane-water interface, where the local water concentration is lower than in the bulk [34,35]. The acquisition of secondary structure at the aqueous-membrane interface by structureless peptides in water has been extensively documented, and it has been ascribed to the favorable environment of this region to provide conditions for intramolecular hydrogen bonding in the case of surface active peptides [13]. Apparently DPPC-LA intermolecular hydrogen bonding was favored at pH 7.4. The higher Tm's (Fig. 5A) can be ascribed to DPPC-LAH domains, possibly at a 1:1 molar ratio, while the remaining of the DPPC molecules would be interspersed with the LA anion. As the effective pK_a_ in the membrane is close to 7.4 [21,36] the local concentrations of LAH and LA forms of the carboxylic acid in the membrane are close. It is conceivable that this heterogeneous distribution of lipid components could be related to the cryo-electron micrographs of Fig. 4 (C, D), especially in the case of the cylindrical shapes observed at pH 7.4.

**Table 2:** T_m_ and ΔT^1/2^ values for DPPC: LAH LUVs in 10 mM Tris-HCl buffer, pH 7.4 and10 mM Na acetate buffer, pH 5.0.

% LAH	pH 5.0		pH 7.4	
	Tm (°C)	ΔT^1/2^	Tm (°C)	ΔT^1/2^
0	41.1	1.49	41.4	1.35
10	41.8	1.89	41.1	1.53
20	42.4	2.65	41.5	1.80
30	43.0	2.65	41.7	2.43
40	45.6	1.18	42.1/42.8	-
50	45.1	1.35	41.9/42.9	-

The analysis presented above clarifies the data for pH 5.0 (Fig. 5B) where thermograms show broadening of the curves for 10, 20, and 30 % LAH, with the curve's maxima shifting to higher temperatures, and subsequently becoming narrower, with maxima at 45.6 and 45.1 °C for 40 and 50 % LAH (Table 2). At this pH, only the LAH form is present in the bilayer, and virtually all added LAH is incorporated (Section 3.1). The thermograms can be interpreted by the formation of 1:1 DPPC: LAH complexes coexisting with pure DPPC domains at lower LAH contents (10, 20 and 30 mole % LAH) and to the bilayer being formed by essentially 1:1 DPPC: LAH complexes at 40 and 50 mole % LA (Fig. 5B), whose phase transition occurs at higher temperature (45.6 °C) due to the strong hydrogen bonding between DPPC and LAH. On this basis, it can be proposed that DPPC bilayers containing high percentages of LAH are tightly packed and would possibly release the fatty acid at a slow rate. Interestingly, most studies of the interaction between carboxylic acids and phosphatidylcholines containing 1,2-saturated acyl chains where strong hydrogen bonding was observed were performed with fatty acids and phospholipids bearing the same chain length [37]. In the present case, the fact that LAH is four carbons shorter than DPPC might lead one to expect more of a disordering effect (loss of cooperativity) in the acyl chain region. Nonetheless, the thermograms indicate that the effect of hydrogen bonding prevails, and the final status is that of a more tightly packed system, whose T_m_ is higher than that of DPPC (Fig. 5B).

### 3.6 Skin permeation of vesicle-incorporated lauric acid

**Fig. 6:**
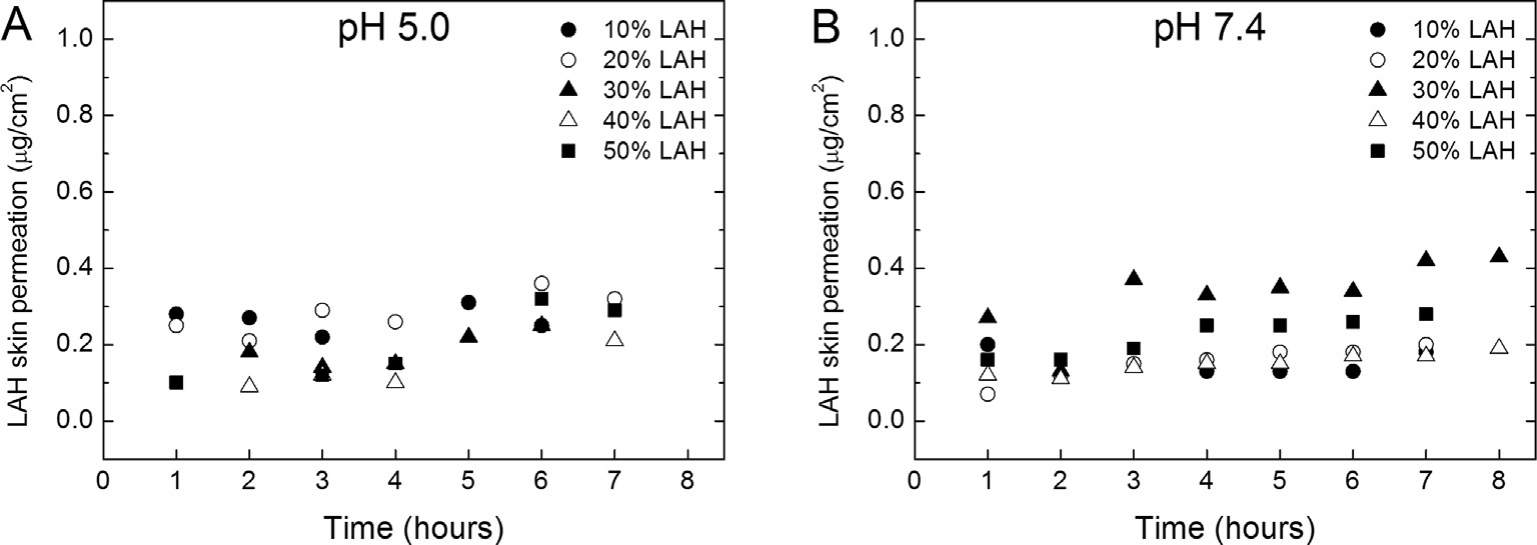
LAH skin permeation (in μg/cm^2^) from DPPC: LAH LUVs as a function of time, in 0.01 M Na^+^ acetate buffer pH 5.0 (A) and 0.01M Tris-HCl buffer, pH 7.4 (B) at several DPPC: LAH molar ratios. The volume of samples in the donor chamber was 0.5 mL and total lipid concentration was 0.5 mM. The receptor chamber was 5 mL. The area of the pig skin was 0.64 cm^2^. The percentages of LAH in DPPC: LAH vesicles were: 10 % (●); 20 % (○); 30 % (▲); 40 % (Δ) and 50 % (■).

The kinetics of LAH permeation, from LUVs of DPPC: LAH, at different molar ratios, through the pig ear skin, was studied at pHs 5.0 and 7.4. Samples from the receptor chamber (see Methods) were taken hourly for 8 h, and the LAH content (in μg/cm^2^) was analyzed (Fig. 6). No LAH, derived from the LAH content of the skin, was detected in the receptor chamber when LAH-free DPPC LUVs were used as control.

At pH 5.0, for samples containing 10, 20 and 30 % of LAH, the amount of permeated LAH reached ca. 0.3 μg/cm^2^ (Fig. 6A). For LUVs containing 40% and 50% LAH the amount of permeated LAH increased with time reaching a maximum between 0.2 and 0.3 μg/cm^2^, respectively (Fig. 6A). At pH 7.4 (Fig. 6B) the same pattern was observed, the initial LAH permeation was between 0.05 and 0.3 μg/cm^2^ after the first hour, at low % LAH, and reached between 0.2 to 0.3 μg/cm^2^ after 6 h for all % LAH, with exception of the sample containing 50 % LAH which reached 0.4 μg/cm^2^.

**Fig. 7:**
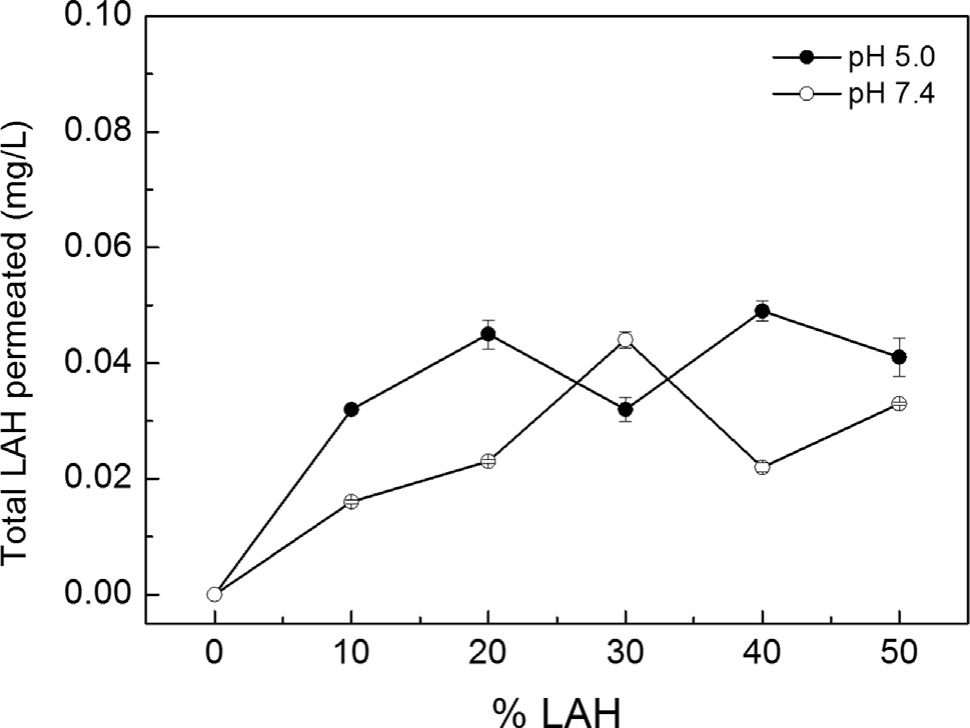
LA skin permeation from DPPC: LAH LUVs, at different % LAH. Total LAH permeated after 6 h (mg/L) as a function of % LAH in LUVS prepared in: (●) 0.01 M Na acetate buffer pH 5.0; and (○) 0.01 M Tris-HCl buffer, pH 7.4. Conditions of the experiment are in the legend of Fig. 6. Each point is the average of three injections. The maximum data spread, for each DPPC: LAH sample, was 8 %.

The total amounts of permeated LAH (mg/L) after 6 h incubation, using LUVs with different % LAH, is shown in Fig. 7. At pH 5.0, the total LAH that in the receptor chamber increased with the LUV % LAH, reaching a maximum for 40 % LAH. Increasing the % LAH in the LUVs from 10 to 50 % only doubled the total permeated LAH (Fig. 7).

At pH 7.4, the maximum permeation was observed with LUVs containing 30 % LAH. Further increases in the % LAH decreased the amount of permeated LAH (Fig. 7). At high %LAH, at pH 7.4, the diffusion of LUVs through the skin could be hampered because of the excess of negative charge on the skin surface [38] leading to the maximum observed in 30 % LAH. The low relative amount of LAH reaching the receptor fluid in the time selected is convenient. Rather, the retention of the active substance, such as LAH, in the stratum corneum, hair follicle, dermis, and epidermis of the skin can be considered to represent cutaneous permeation [39].

**Fig. 8:**
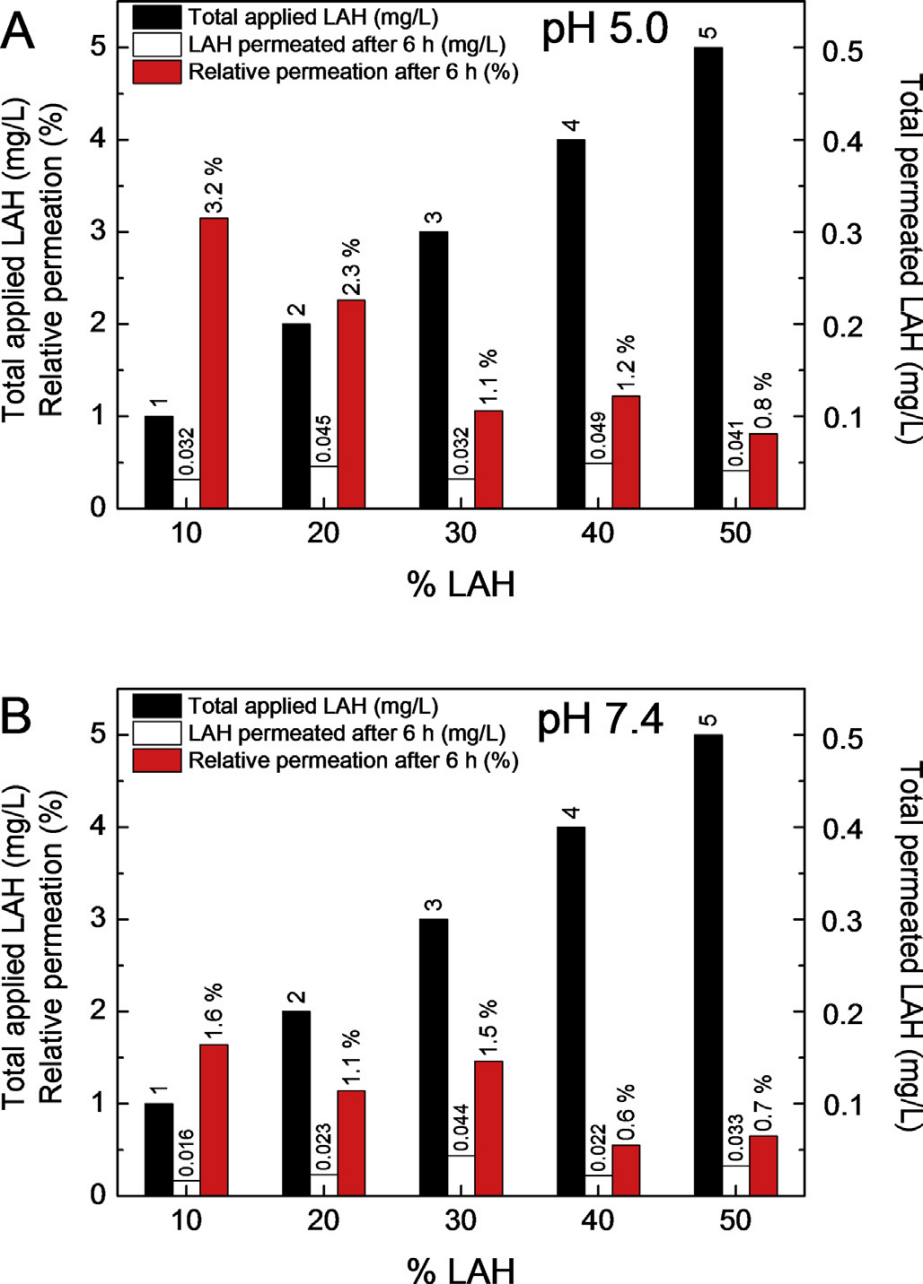
LA skin permeation data from DPPC: LAH vesicles at several % LAH. (■) Total applied LAH (mg/L), (□) Total LAH permeated after 6 h (mg/L) and () % Relative permeation of LAH after 6 h from DPPC: LAH vesicles in (A) 0.01 M Na acetate buffer pH 5.0 and (B) 0.01 M Tris-HCl buffer, pH 7.4.

In Fig. 8, we compare our data of LAH permeation through pig ear skin. The amount of LA recovered in the receptor liquid after 6 h is also plotted vs the % LAH and can be compared the initial amount of LAH in all samples studied, at pH 5.0 and 7.4.

For LUVs containing from 10 to 50 % LAH, the percentage of permeated LAH, was small at both pHs. Even increasing the total amount of LAH from 1 mg/mL to 5 mg/mL, less than 0.05 mg/mL permeated the skin for all vesicle's compositions. At pH 5.0, after 6 h, for LUVs with 10 % and 50 % LAH, the percentages of permeated LAH were 3.2 % and 0.8 %, respectively (Fig. 8A). At pH 7.4, ca. 1.6 % of LAH permeated the skin for LUVS with 10 % LAH and 0.7 % for those with 50 % LAH (Fig. 8B). In conclusion, the increase in the total amount of LAH in the donor liquid did not lead to a proportional increase of LAH in the receptor chamber.

With respect to LAH retention in the skin, Blank and Gould, using ^14^C labeled LAH, demonstrated that LAH dissolved in buffered solutions, at different pH's, penetrates in the human skin dermis and that the LAH penetration is higher as the pH decreases from 8.5 to 7.5 [40]. They pointed out that they could not perform the experiments below pH 7.5 due to the flocculation of the acid form of LAH. Our results show that LAH permeation through the skin, from LUVs of DPPC: LAH, is observed at pHs 5.0 and 7.4 allowing to bypass the insolubility problem of LAH delivery to the skin. However, the skin permeation of LAH is low, as desired for a drug that has to penetrate the skin and have no effect at the systemic level.

LAH-containing LUVs allow the preparation of acid pH stable formulations containing high LAH concentrations, high stability, thus avoiding the flocculation of LAH. This is an exciting result, since in the treatment of acne vulgaris the action of lauric acid on the hair follicle rather than skin permeation has to be considered [5,41].

## 4 Conclusions

Here we described a detailed physicochemical characterization of the potential delivery system consisting of DPPC: LAH vesicles. SAXS and cryo-TEM showed that unilamellar vesicles co-existed with different populations of oligo- or multilamellar DPPC: LAH-containing LUVs and that closed and impermeable vesicles were formed containing an aqueous inner compartment. DSC studies showed that the charged and uncharged forms of lauric acid interact in different manners with the DPPC bilayer and that, at pH 7.4, at least two phases coexist, one possibly being enriched with LAH. At pH 5.0, a hydrogen bonded-mediated complex between DPPC and LAH may be responsible for a system with higher phase transition temperature. The small amounts of LAH detected in the receptor fluid of Franz cell is suggestive of the ability of LAH-containing DPPC vesicles to deliver the fatty acid to the skin. As we demonstrated that the LAH-containing lipid vesicles incorporate water soluble probes, the DPPC: LAH vesicle system may serve, at the same time, to deliver the antimicrobial lauric acid and a water-soluble active compound to the skin.

## Declarations

### Author contribution statement

I. Midea Cuccovia: Conceived and designed the experiments; Analyzed and interpreted the data; Contributed reagents, materials, analysis tools or data; Wrote the paper.

L. Farkuh: Conceived and designed the experiments; Performed the experiments; Analyzed and interpreted the data; Wrote the paper.

P. T. Hennies: Conceived and designed the experiments.

C. Nunes, M. A. Segundo, L. Barreiros, P. L. Oseliero Filho, C. L. P. Oliveira, A. Cassago, R. V. Portugal, R. A. Muramoto, G. P. B. Carretero: Performed the experiments.

S. Reis: Analyzed and interpreted the data.

S. Schreier: Analyzed and interpreted the data; Wrote the paper.

H. Chaimovich: Analyzed and interpreted the data; Contributed reagents, materials, analysis tools or data; Wrote the paper.

### Funding statement

L. Farkuh thanks Ph.D. Bárbara Bianca Gerbelli (IF-USP), CNPq, FAPESP (Proc. 2013/08166-5), Santander Universidades (COPGRAD.01 - 044/2014). I.M.Cuccovia thanks the National Council for Scientific and Technological Development (CNPq – 465259/2014-6), the Coordination for the Improvement of Higher Education Personnel (CAPES), the National Institute of Science and Technology Complex Fluids (INCT-FCx), and the São Paulo Research Foundation (FAPESP – 2014/50983-3). We acknowledge the Brazilian Nanotechnology National Laboratory (LNNano), CNPEM, for the use of cryo-TEM facilities. This work was supported by The European Union (FEDER funds) and National Funds (FCT/MEC, Fundação para a Ciência e a Tecnologia and Ministério da Educação e Ciência) under the Partnership Agreement PT2020 UID/QUI/50006/2013 - POCI/01/0145/FEDER/007265. L. Barreiros thanks FCT and POPH (Programa Operacional Potencial Humano) for her Post-Doc grant (SFRH/BPD/89668/2012). C. Nunes thanks FCT for her Investigator Grant (IF/00293/2015). G.P.B.Carretero acknowledges the Programa CAPES: INCT -Institutos Nacionais de Ciência e Tecnologia (Proc. 88887.137085/2017-00), R.A. Muramoto acknowledges CNPq. I.M. Cuccovia, H. Chaimovich, and S. Schreier are research fellows of CNPq.

### Competing interest statement

The authors declare no conflict of interest.

### Additional information

No additional information is available for this paper.
